# Control of the Nitrogen Isotope Composition of the Fungal Biomass: Evidence of Microbial Nitrogen Use Efficiency

**DOI:** 10.1264/jsme2.ME18082

**Published:** 2018-12-15

**Authors:** Kazuki Shinoda, Midori Yano, Muneoki Yoh, Makoto Yoshida, Akiko Makabe, Yohei Yamagata, Benjamin Z. Houlton, Keisuke Koba

**Affiliations:** 1 United Graduate School of Agricultural Science, Tokyo University of Agriculture and Technology Tokyo, 183–8509 Japan; 2 Institute of Agriculture, Tokyo University of Agriculture and Technology Tokyo, 183–8509 Japan; 3 Center for Ecological Research, Kyoto University Shiga, 520–2113 Japan; 4 Project Team for Development of New-generation Research Protocol for Submarine Resources, Japan Agency for Marine-Earth Science and Technology Kanagawa, 237–0061 Japan; 5 Department of Land Air and Water Resources, University of California Davis, California 95616 USA

**Keywords:** *Aspergillus oryzae*, soil microbial biomass, nitrogen mineralization, nitrogen use efficiency, δ^15^N

## Abstract

Changes in ^15^N/^14^N in the soil microbial biomass during nitrogen (N) mineralization have been hypothesized to influence ^15^N/^14^N in soil organic matter among ecosystem sites. However, a direct experimental test of this mechanism has not yet been performed. To evaluate the potential control of microbial N mineralization on the natural N isotope composition, we cultured fungi (*Aspergillus oryzae*) in five types of media of varying C:N ratios of 5, 10, 30, 50, and 100 for 4 d, and tracked changes in δ^15^N in the microbial biomass, NH_4_^+^, and dissolved organic N (DON: glycine) over the course of the experiment. High rates of NH_4_^+^ excretion from *A. oryzae* were accompanied by an increase in δ^15^N in the microbial biomass in low C:N media (*i.e*., C/N<30). In contrast, NH_4_^+^ was strongly retained in higher C/N treatments with only minor (*i.e*., <1 ‰) changes being detected in δ^15^N in the microbial biomass. Differences in δ^15^N in the microbial biomass were attributed to the loss of low-δ^15^N NH_4_^+^ in low, but not high C/N substrates. We also detected a negative linear correlation between microbial nitrogen use efficiency (NUE) and Δ^15^N (δ^15^N-biomass–δ^15^N-glycine). These results suggest an isotope effect during NH_4_^+^ excretion in relatively N-repleted environments in which microbial NUE is low, which may explain the vertical patterns of organic matter δ^15^N in soil profiles.

Soil microbial processes strongly regulate the terrestrial nitrogen (N) cycle. The microbial decomposition of soil organic matter (SOM) aids in the mineralization of N in plants, which maintains primary productivity (39, 53) and influences the magnitude of denitrification and nitrification (23, 55). Increasing levels of atmospheric CO_2_ and elevated N deposition rates markedly affect the N cycle (19, 30, 51), partly by changing the quantity and quality of SOM (61). Therefore, it is important to clarify the relationships between soil microbial processes and N and carbon (C) availabilities in order to predict changes in terrestrial ecosystem N cycling.

The soil microbial biomass (SMB) accounts for a significant fraction of the soil N pool (64) and contributes to SOM production and consumption (40, 49). For example, ^13^C and ^15^N NMR analyses revealed that SMB in soil minerals contributed to SOM pools (21, 33). The fungal biomass is a demonstrated precursor of SOM formation (9, 17, 31), *e.g*. the dominant pathway (62%) through which C enters the SOM pool, exceeding C input via leaf litter and fine root turnover, with potentially similar patterns for N (22).

The natural abundance of ^15^N/^14^N (δ^15^N; δ^15^N is expressed as ^15^N/^14^N_sample_/^15^N/^14^N_standard_–1, where ^15^N/^14^N_standard_ is atmospheric N_2_ and treated in units mil^−1^ [‰]) was previously used to examine relationships among SOM and SMB (13). While whole-soil δ^15^N patterns are driven by variations in the δ^15^N of inputs and the balance among leaching and isotopic fractionating gaseous N loss over time (28, 29, 37, 45), the within soil profile δ^15^N is a product of the downward transport of δ^15^N, microbial processing, and variations in δ^15^N in root and litter inputs. The δ^15^N of surface (*i.e*., ~top 10 cm) SOM markedly varies among ecosystems, from −7.8‰ in acidic tundra (3) to as high as 22.0‰ in South Africa (13); however, the mechanisms responsible for these variations remain unclear (13, 26).

A primary factor contributing to δ^15^N-SOM patterns may be the rates of SOM decomposition (11, 35, 38, 56, 59). Kramer *et al*. (38) reported a strong positive relationship between δ^15^N-SOM and an index for SOM decomposition (Aliphatic/O-Alkyl), and Sollins *et al*. (56) demonstrated that δ^15^N-SOM increased with greater densities in the SOM fraction. These findings imply that high δ^15^N in SMB-derived compounds is either incorporated into SOM, ^15^N-depleted SOM is lost during decomposition, or both.

δ^15^N-SMB, which is measured using a chloroform-fumigation-extraction procedure (14, 62), was shown to be higher than δ^15^N-SOM in most soils, with the level of ^15^N enrichment of SMB (Δ^15^N=δ^15^N-SMB–δ^15^N-SOM) varying widely across soils (Table 1). Furthermore, Δ^15^N increases with decreasing soil C/N (16, 41). Dijkstra *et al*. (16) hypothesized that the ^15^N/^14^N of SMB is controlled by the excretion of ^15^N-depleted ammonia from the microbial biomass. In this case, δ^15^N-SMB is expected to increase when soil microbes actively mineralize organic N at low C/N (N sufficient conditions), while δ^15^N-SMB shows markedly less enrichment when net N is immobilized (incorporate) at high C/N (N limited conditions).

^15^N/^14^N enrichment in organisms vs. substrates is common. Animals are commonly enriched in ^15^N by ~3–5‰ vs. food sources, with the δ^15^N of excreted compounds (NH_4_^+^, urea, and urine) being lower than that of the biomass in cows (57), guppies (44), and zooplankton (6). In contrast, less direct evidence is available for microbial ^15^N enrichment vs. N sources; however, several observations point to this possibility. For example, the δ^15^N of bacteria grown on alanine as a single nitrogen substrate was 3.6‰ higher than that of alanine (42). Yamaguchi *et al*. (65) cultured bacteria, fungi, and archaea with casamino acids, and observed ^15^N enrichment of the biomass in the order of 0.6±0.2, 3.6±0.2, and 5.0±1.0‰, respectively, vs. substrate δ^15^N. In a controlled culture experiment, Collins *et al*. (10) demonstrated an increase in δ^15^N in bacterial cells during N mineralization.

Previous studies suggested that N availability and the degree of N limitation are primary factors contributing to the level of microbial ^15^N enrichment, which may be expressed through the concept of nitrogen use efficiency (NUE). NUE is defined as the ratio of N incorporation into the microbial biomass to organic N uptake (46) and is expected to decrease when soil microbes actively mineralize organic N at low C/N. In contrast, NUE increases when net N is immobilized at high C/N owing to microbial N limitations. Thus, we hypothesize that NUE controls Δ^15^N and, thus, δ^15^N-SOM patterns.

We herein investigated the mechanisms underlying the ^15^N enrichment of fungi via a controlled culture experiment, and measured δ^15^N in the biomass, substrates, and NH_4_^+^. Fungi were cultured in media with different C:N ratios to examine the hypothesis that NUE is a primary factor responsible for the level of isotopic expression. In the present study, we provide direct evidence to show that the δ^15^N of fungi is influenced by NUE.

## Materials and Methods

### Experimental design

*Aspergillus oryzae* (RIB40, NFRI1599), a well-studied, cosmopolitan strain of fungi, was used in the present study. Five milliliters of *A. oryzae* (*ca*. 8×10^7^ cells) was inoculated into 500-mL flasks with 250 mL of medium. Cultures were incubated at 27°C with shaking (180 rpm) for 96 h. Porous silicon plugs were used to maintain aerobic conditions. We used glycine and glucose as the sole C and N sources to adjust the C:N ratio of medium. Five types of media with C:N ratios of 5, 10, 30, 50, and 100 (hereafter CN5, CN10, CN30, CN50, and CN100, respectively) contained glycine and D(+)-glucose at the following amounts: 3.26 and 4 g L^−1^, 1.63 and 5.2 g L^−1^, 0.5 and 5.6 g L^−1^, 0.25 and 4.8 g L^−1^, and 0.2 and 7.9 g L^−1^, respectively. The actual initial C:N ratios of media were 5.4±0.1, 11.1±0.2, 31.3±0.5, 52.5±2.0, and 103.6±3.8 (mol mol^−1^; means±S.D.). All media contained 0.5 g L^−1^ MgSO_4_·7H_2_O, 0.07 g L^−1^ CaCl_2_·2H_2_O, 0.01 g L^−1^ FeSO_4_·7H_2_O, 4.65 mg L^−1^ MnSO_4_·5H_2_O, 5.0 mg L^−1^ ZnSO_4_·7H_2_O, and 1.0 mg L^−1^ CoCl·6H_2_O. pH was adjusted to between 5.7 and 5.9 by adding 0.92 g L^−1^ K_2_HPO_4_ and 6.86 g L^−1^ KH_2_PO_4_. Media were filtered through pre-sterilized membrane filters (pore size of 0.22 μm; Stericup and Steritop, Millipore, Burlington, MA, USA), and filtrates were poured into pre-combusted (450°C, 4 h) 500-mL flasks. A total of 1.0 mg L^−1^ FeSO_4_·7H_2_O was added to medium without sterilization because of its oxidization during autoclaving as well as its precipitation. We had 3 replicates, except for CN 5 which had 4 replicates.

Samples were collected every 24 h for a total of 4 d. Five milliliters of media, including the biomass, was collected using a 5-mL pipette and filtered through pre-combusted (450°C, 4 h) glass fiber filters (GF/F; Whatman, Tokyo, Japan) with a pore size of 0.22 μm (Steriflip Filter Unit, Millipore), while all media in the flasks were filtered with a membrane filter (pore size of 0.22 μm; Stericup and Steritop, Millipore) to completely recover the biomass in the flask at 96 h. The filtrate was frozen for later analyses. The biomass on the filter was washed 4 times with 5 mL (total of 20 mL) of 0.9% (w/v) NaCl solution, separated from the filter, transferred to a microtube, and freeze-dried. The growth of *A. oryzae* was calculated from the freeze-dried biomass weight.

### Data collection and analysis

The concentrations of NH_4_^+^ and NO_3_^−^ ([NH_4_^+^] and [NO_3_^−^], respectively) in filtrates were assessed by colorimetric methods using an autoanalyzer (QuAAtro2-HR; BL-TEC, Tokyo, Japan). [NO_3_^−^] was not detected in any samples (the detection limit of 0.1 μM for the sum of NO_2_^−^ and NO_3_^−^). The concentrations of dissolved organic C ([DOC]) and total dissolved N ([TDN]) were measured using a TOC analyzer with a TN unit (TOC-L/TNM-L; Shimadzu, Kyoto, Japan). The concentration of dissolved organic N ([DON]) was calculated as follows: (DON)=(TDN)–(NH_4_^+^). The C:N ratio of medium (DOC/TDN) was calculated as DOC/TDN=(DOC)/(TDN).

The recovery % of N during the incubation was calculated as follows: Recovery N (%)=([TDN]+[biomass N])/(TDN_initial_)×100, where (TDN_initial_) is the concentration of TDN at 0 h and (biomass N) is the concentration of the biomass N. Microbial NUE was calculated as ([DON_consumption_]–[NH_4_^+^_production_])/(DON_consumption_), where (DON_consumption_) represents the difference between initial DON and DON concentrations over the course of the experiment, and (NH_4_^+^_production_) is the difference between NH_4_^+^ concentrations and vs. initial NH_4_^+^ measured.

The concentrations of biomass carbon ([biomass C]) and (biomass N) and the δ^15^N values of the biomass (δ^15^N-biomass) were measured using an elemental analyzer (EA1112; Thermo Fisher Scientific, Yokohama, Japan) interfaced with an isotope ratio mass spectrometer (Delta-XP; Thermo Fisher Scientific). Calibrated in-house standards, such as DL-alanine (δ^15^N=−1.7‰), glycine (δ^15^N=10.0‰), and L-histidine (δ^15^N=−8.0‰), were used to obtain the calibration curve to correct the measured isotopic values. The average standard deviations for a replicate analysis of individual samples were ±0.4‰ for δ^15^N after blank corrections. The biomass C/N (mol mol^−1^) was calculated as biomass C/N=(biomass C)/(biomass N).

The δ^15^N values of NH_4_^+^ (δ^15^N-NH_4_^+^) were measured for samples with CN5 and CN10 only after 48 h with sufficient concentrations of (NH_4_^+^) that allowed for the assessment of δ^15^N-NH_4_^+^. δ^15^N-NH_4_^+^ was measured using the methods described by Koba *et al*. (36). In brief, NH_4_^+^ was concentrated on the pre-combusted (450°C, 4 h) glass fiber filter (GF/D, diameter of 1 cm, Whatman) using the diffusion method (27), and concentrated NH_4_^+^ was then oxidized to NO_3_^−^ using persulfate (34). The δ^15^N of converted NO_3_^−^ from NH_4_^+^ was measured using the denitrifier method (4, 54) with an isotope ratio mass spectrometer (IRMS; 20–22: Sercon, Cheshire, UK) coupled with an autosampler (GX-271: GILSON, Middleton, WI, USA) and CryoPrep (Sercon) equipped with a gas chromatograph (GC-4000, GL Sciences, Tokyo, Japan). The denitrifying bacterium, *Pseudomonas aureofaciens* (ATCC#13985), which lacks nitrous oxide reductase activity, was used to convert NO_3_^−^ into N_2_O gas before the isotope analysis. The isotopic standards, USGS25 (δ^15^N=−30.4‰), USGS26 (δ^15^N=53.7‰), and IAEA N2 (δ^15^N=20.3‰), were used for data calibration. The average standard deviations from the replicate analysis of individual samples were large (±1.4‰) for δ^15^N after blank corrections, and this was attributed to samples with low (NH_4_^+^) (CN10 at 48 h). The average standard deviations from the replicate analysis were ±0.1‰ without these low (NH_4_^+^) samples after blank corrections.

The δ^15^N of glycine at 0 h (δ^15^N-glycine) and δ^15^N of TDN (δ^15^NTDN) were measured via the denitrifier method, in which total N was digested to NO_3_^−^ using the persulfate method (36). Calibrated in-house standards of DL-alanine (δ^15^N=−1.7‰), glycine (δ^15^N=10.0‰), and L-histidine (δ^15^N=−8.0‰) were used for the calibration. The average standard deviations for the replicate analysis of individual samples were ±0.2‰ for δ^15^N after blank corrections. Δ^15^N was calculated as the difference between δ^15^N-biomass and δ^15^N-glycine (Δ^15^N=δ^15^N-biomass–δ^15^N-glycine).

In some cases, (TDN) was lower than (NH_4_^+^) because of the inaccurate assessment of high (NH_4_^+^), and we were unable to calculate δ^15^N-DON with the concentrations and δ^15^N data of TDN and NH_4_^+^. On the other hand, the concentration of Gly_used_, glycine utilized by *A. oryzae*, (Gly_used_), was calculated as (Gly_used_)=(biomass N)+(NH_4_^+^). The δ^15^N of Gly_used_ (δ^15^N-Gly_used_) was then calculated with the concentrations and δ^15^N values of biomass N and NH_4_^+^ as δ^15^N-Gly_used_= δ^15^N_microbe_×f_microbe_+δ^15^N-NH_4_^+^×f_NH4+_ and f_microbe_+f_NH4+_=1, where f_NH4+_ is the ratio of the concentration of initial NH_4_^+^ to TDN and f_microbe_= 1–f_NH4+_. δ^15^N-Gly_used_ was not calculated in CN30, CN50, and CN100 because the concentrations of NH_4_^+^ were too low to measure δ^15^N-NH_4_^+^. The standard deviations of δ^15^N-Gly_used_ were evaluated using the Monte Carlo method described by Koba *et al*. (37).

Statistical analyses were conducted using R software (R version 3.4.2, R core Team [2017]). An α level of 0.05 was considered to indicate significance. The Student’s *t*-test was used to investigate whether concentrations or δ^15^N values were significantly different between (DOC)/(TDN) and biomass C/N on each d in all treatments, between δ^15^N-biomass at 96 h and initial δ^15^N-glycine (0.1±0.3‰) in all treatments, and between δ^15^N-Gly_used_ in CN5 and CN10 and initial δ^15^N-glycine (0.1±0.3‰) on each d. The paired Student’s *t*-test was used to clarify whether concentrations or δ^15^N values were significantly different between δ^15^N-biomass at 24 and 96 h in all treatments, and between δ^15^N-NH_4_^+^ and δ^15^N-biomass on each d in CN5 and CN10. Pearson’s correlation test was used to examine the relationship between (DOC)/(TDN).

## Results

### The microbial biomass and carbon and nitrogen dynamics

**Fig. 1:**
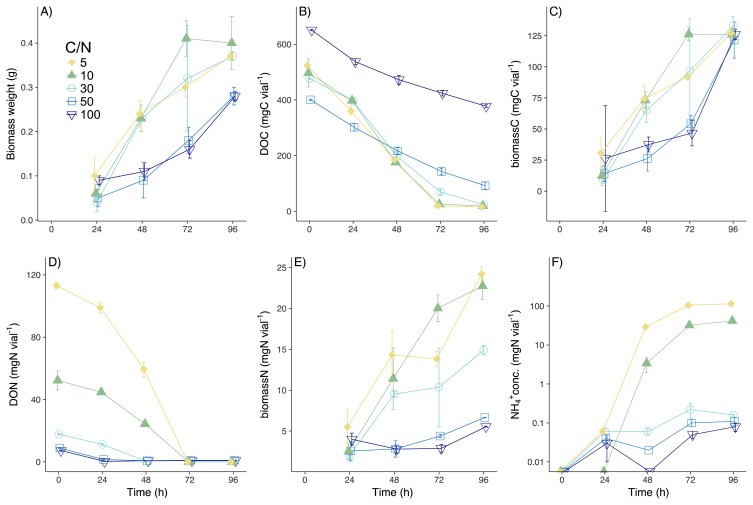
Changes in biomass weights (A), concentrations of DOC (B), biomass C (C), DON (D), biomass N (E), and NH_4_^+^ (F) at different C:N treatments. Symbols represent mean values and error bars represent SD. Regarding samples without error bars, the SD was smaller than the symbols.

**Fig. 2:**
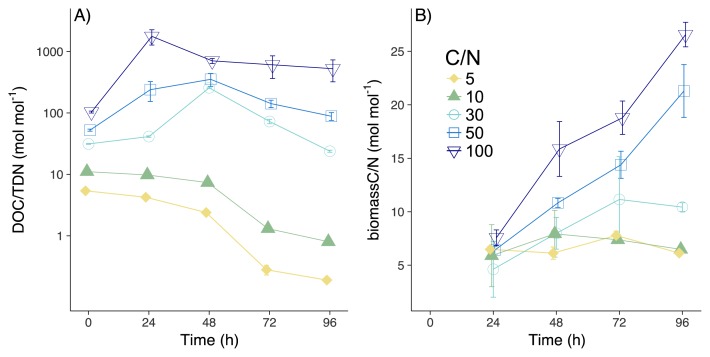
Changes in DOC/TDN (A) and biomass C/N (B) at different C:N treatments. Symbols represent mean values and error bars represent SD. Regarding samples without error bars, the SD was smaller than the symbols.

As *A. oryzae* grew (Fig. 1A), the majority of DOC was consumed by 96 h (97.1±0.1, 96.4±0.1, 95.1±0.3, 77.1±2.1, and 41.9±0.6% [mean±SD] in CN5, CN10, CN30, CN50, and CN100, respectively; Fig. 1B), thereby increasing biomass C over the course of the experiment (Fig. 1C; 24.9±1.0, 25.1±0.9, 29.2±1.6, 39.2±2.6, and 46.3±1.6% of consumed DOC were assimilated in CN5, CN10, CN30, CN50, and CN100, respectively). More than 90% of the DON substrate was consumed in CN30, CN50, and CN100, with DON concentrations decreasing to below the limit of detection in CN5 and CN10 at 72 and 96 h (Fig. 1D). Large amounts of DON were incorporated in biomass N by 96 h (Fig. 1E; 21.4±1.2, 41.1±3.1, 87.6±1.6, 85.0±6.2, and 87.2±5.0% for CN5, CN10, CN30, CN50, and CN100, respectively). Consumed DON was mineralized to NH_4_^+^ in CN5 and CN10 (92±2 and 57±10% of consumed DON, respectively), while less than 2% of consumed DON was mineralized to NH_4_^+^ in other treatments (Fig. 1F). Nitrate concentrations were below the limit of detection in all treatments. In CN5 and CN10, biomass C/N was significantly higher than DOC/TDN (except for 24 and 48 h in CN10; Fig. 2A and B, Table S1; *P*<0.001). On the other hand, DOC/TDN was significantly higher than biomass C/N in CN30, 50, and 100 (Fig. 2A and B, Table S1; *P*<0.01). Moreover, biomass C/N in CN50 and CN100 increased to 21.3±2.5 and 26.6±1.2, respectively, values that were significantly higher than the C/N of soil fungi (4–17) when incubated in medium C/N of 3.9 (47). Although high C/N ratios are not common, fungi may respond to a stoichiometric imbalance by increasing the storage of C in cells (*e.g*. in lipids [47]), thereby increasing C/N. Another possibility is that the microbial biomass incorporated the degraded necromass at low N concentrations, resulting in the high C/N observed in the total biomass.

The recovery of N was more than 70% in CN5 and CN10, but was lower (40–62%) from 24 to 72 h in CN30, CN50, and CN100 (except at 96 h; Fig. S1). This low recovery of N (less than 100%) was attributed to the underestimation of biomass N (the largest N fraction in this experiment) between 24 and 72 h. We collected the microbial biomass with large membrane filters (see the Methods section) at 96 h, but used a 5-mL pipette with an aperture that was not sufficiently large to collect the same rate of microbial biomass for 250 mL medium until 72 h. Underestimated biomass N (%) may be defined as ([expected biomass N]/[measured biomass N])×100, where (expected biomass N)=(DON consumption)–(NH_4_^+^ production). Between 24 and 72 h, underestimated biomass N was between 36 and 60%, while only 10–12% of biomass N was underestimated at 96 h in CN30, CN50, and CN100. We measured biomass N at 96 h at the end of the incubation more accurately than at other time points because we collected all of the microbial biomass left in media using large membrane filters (see the Materials and Methods section), while we collected microbial biomass with a small ratio for 5 mL medium solution using the 5-mL pipette until 72 h. Since DON was nearly consumed by 48 h in CN30 and by 24 h in CN50 and CN100 (Fig. 1D) and less than 2% of consumed DON was mineralized to NH_4_^+^, we assumed that the concentrations of biomass N at 48 and 72 h in CN30 and between 24 and 72 h in CN50 and CN100 were similar to that of biomass N at 96 h in each CN treatment. After we recalculated biomass N based on this assumption, the recovery of N in CN30, CN50, and CN100 at 24, 48, and 72 h increased to 74–92%.

### Changes in δ^15^N

**Fig. 3:**
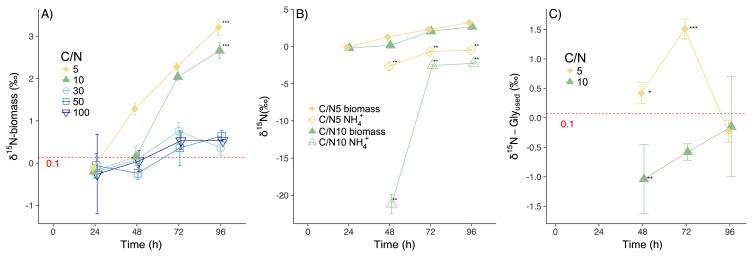
A) Changes in δ^15^N-biomass in CN5 and CN10. The red dotted line represents δ^15^N-Glycine (0.1±0.3‰). Asterisks (***) represent a significant difference (*P*<0.001) between δ^15^N-Glycine and δ^15^N-biomass at 96 h based on the Student’s *t*-test. B) Changes in δ^15^N-biomass and δ^15^N-NH_4_^+^ in CN5 and CN10. In CN30, CN50, and CN100, the concentration of NH_4_^+^ was too low to measure δ^15^N-NH_4_^+^. Asterisks (**) represent a significant difference (*P*<0.01) between δ^15^N-NH_4_^+^ and δ^15^N-biomass based on the paired Student’s *t*-test. C) Changes in δ^15^N-Gly_used_ in CN5 and CN10. The red dotted line represents δ^15^N-Glycine. Asterisks represent a significant difference between δ^15^N-Gly_used_ and δ^15^N-Glycine based on the Student’s *t*-test (*, **, *** indicate *P*<0.05, *P*<0.01, *P*<0.001, respectively). Symbols represent mean values and error bars represent SD. Regarding samples without error bars, the SD was smaller than the symbols.

δ^15^N-biomass by 24 h was −0.1±0.5 and −0.2±0.2‰ in CN5 and CN10, respectively; a significant increase in δ^15^N-biomass between 3.1±0.3 and 2.9±0.0‰ was apparent by 96 h (Fig. 3A, Table S2, *P*=0.001 and *P*<0.001). δ^15^N-biomass at 96 h was significantly higher than δ^15^N-glycine (0.1±0.3‰) (Fig. 3A, Table S2, *P*<0.001 and *P*<0.001), and Δ^15^N was 3.1±0.5 and 2.7±0.2‰ in CN5 and CN10, respectively. On the other hand, in CN30 and CN100, δ^15^N-biomass was not significantly different between 24 h (−0.2±0.1 and −0.3±0.9‰) and 96 h (0.4±0.2 and 0.5±0.0‰) (Fig. 3A, Table S2, *P*=0.076 and *P*=0.279). In CN50, δ^15^N-biomass was significantly different between 24 h (−0.1±0.3‰) and 96 h (0.6±0.1‰) (Fig. 3A, Table S2, *P*=0.037); however, this difference (0.7±0.2‰) was markedly smaller than that observed for CN5 and CN10. In CN30, CN50, and CN100, δ^15^N was not significantly different between glycine and biomass N at 96 h (Fig. 3A, Table S2, *P*=0.358, *P*=0.162, *P*=0.227, respectively), and Δ^15^N (0.4±0.2, 0.7±0.1, 0.6±0.0‰, respectively) was smaller than those for CN5 and CN10.

δ^15^N-NH_4_^+^ was below the limit of quantification in CN30, CN50, and CN100 (Fig. 3B). In CN5 and CN10, δ^15^N-NH_4_^+^ was significantly lower than δ^15^N-biomass (Table S3); the differences between them at 48, 72, and 96 h were 3.9±0.6, 2.7±0.5, and 3.5±0.6‰ in CN5, and 21.4±1.1, 4.6±0.2, and 4.9±0.2‰ in CN10, respectively. Among δ^15^N-Gly_used_ and δ^15^N-glycine (0.1±0.3‰), significant differences were observed at 48 and 72 h in CN5 and at 48 h in CN10 (Fig. 3C, Table S4). However, differences between δ^15^N-Gly_used_ and δ^15^N-glycine (average of δ^15^N-Gly_used_–average of δ^15^N-glycine) remained small at 48, 72, and 96 h (+0.35, +1.44, and −0.30 in CN5, and −1.11, −0.65, and −0.22 in CN10, respectively).

### NUE and Δ^15^N

**Fig. 4:**
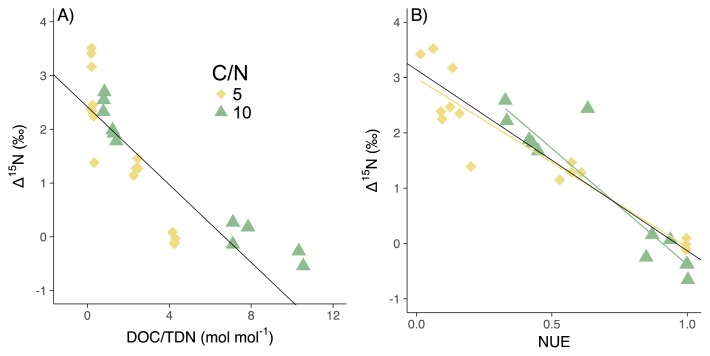
A) Relationship between DOC/TDN and Δ^15^N in CN5 and CN10. The black line represents the linear regression line of two treatments (Δ^15^N=−0.4*DOC/TDN+2.4, *R*^2^=0.86, *P*<0.00). B) Relationship between NUE and Δ^15^N in CN5 and CN10. The black line represents the linear regression line of two treatments (Δ^15^N=−3.3*NUE+3.1, *R*^2^=0.76, *P*<0.001). The yellow and green lines represent the linear regression lines of CN5 and CN10 (Δ^15^N=−3.0*NUE+3.0, *R*^2^=0.89, *P*<0.001 and Δ^15^N=−4.2*NUE+3.8, *R*^2^=0.86, *P*<0.001).

Microbial NUE changed according to the nutrient imbalance (Fig. S2). NUE was examined in CN5 and CN10 only because of the low recovery of biomass N in CN30, CN50, and CN100 (Table S1). A negative linear correlation was observed between DOC/TDN and Δ^15^N (Fig. 4A) and NUE and Δ^15^N (Fig. 4B). Linear regression lines for CN5 (Δ^15^N=−3.0×NUE+3.0 [*R*^2^=0.89, *P*<0.001]) and CN10 (Δ^15^N=−4.2×NUE+3.8 [*R*^2^=0.86, *P*<0.001]; Fig. 4B) had similar slopes, but slightly different intercepts (*P*=0.06 for slopes and *P*<0.0001 for intercepts by an analysis of co-variance; Fig. 3B); however, the difference in intercepts was smaller than the error for isotope measurements (±0.6‰). The isotopic fractionation of NH_3_ volatilization was negligible because the pH of all media were lower than 7 through the experiment. The data obtained are shown in Table S5.

## Discussion

### Mechanism of ^15^N enrichment

We tested hypotheses that the δ^15^N of extracted substrates explains the patterns of microbial biomass enrichment, with implications for the pattern and regulation of surface SOM δ^15^N across ecosystems. We predicted that the δ^15^N of NH_4_^+^ may be depleted more at higher levels of N available to microbes (*i.e*., low C/N), with N functioning as more of an excess nutrient and, thus, being more rapidly mineralized from organic substrates. The present results confirmed these hypotheses; the mineralization (excretion of NH_4_^+^) of DON by *A. oryzae* in CN5 and CN10 (Fig. 1D and F) was accompanied by increases in δ^15^N-biomass over time (Fig. 3A), revealing that N supplies from DON exceeded N demands at low C/N levels. Moreover, *A. oryzae* retained DON in CN30, CN50, and CN100 without any detectable changes in δ^15^N-biomass, thereby demonstrating the importance of N availability in assessing the loss of low-δ^15^N NH_4_^+^ from microbes. According to the mass balance, any elevation in δ^15^N in the microbial biomass vs. substrates must be balanced by a loss of low δ^15^N-NH_4_^+^. δ^15^N-NH_4_^+^ was significantly lower than δ^15^N-biomass in CN5 and CN10 (Table S3), with prominent isotopic excursions that varied between 2.0 and 22.7‰ (Fig. 3B). This level of isotopic depletion is similar to observations for cows (7.3‰; 57), fish (23.3–31.8‰; 44), and plankton (3‰; 6).

Mechanistically, our results revealed that the loss of ^15^N-dpeleted NH_4_^+^ was the principal determinant of fungal biomass δ^15^N, which was very important when N was high (*i.e*., low C/N substrate ratios) (Fig. 1F, 3A, and B). A small isotope effect was observed during DON assimilation by *A. oryzae*, with only minor Δ^15^N values observed for C/N ratios in excess of 30 (Fig. 3A, Table S2). In contrast, Δ^15^N increased over time in CN5 and CN10 (Fig. 3A, Table S2), with little evidence for changes in δ^15^N-Gly_used_ (Fig. 3C, Table S4). These results revealed negligible isotope fractionation during DON uptake by fungi, as reported previously for mycorrhiza (18). The negative correlation between DOC/TDN and Δ^15^N in CN5 and CN10 (*R*=−0.88, *P*<0.001, Fig. 4A), which is similar to the negative correlation between the soil C:N ratio and soil microbial Δ^15^N (16, 41), supports the hypothesis that the mineralization of low δ^15^N-NH_4_^+^ is the primary driver of microbial δ^15^N (16).

While the absorption of isotopically depleted δ^15^N-NH_4_^+^ by *A. oryzae* may decrease, δ^15^N-biomass may, in principle, decrease δ^15^N in the microbial biomass (−14.1±0.8‰ [60]); however, this was not the case in the present study. Although DON and NH_4_^+^ absorption both occur in ecosystems (53), *A. oryzae* did not appear to use excreted NH_4_^+^ due to C limitations at low C/N ratios; the low concentrations of NH_4_^+^ in CN30, CN50, and CN100 may have strongly limited the importance of this N source in the present study (Fig. 1F, 2A, and B).

We used glycine as the sole N source because it accounts for a large fraction of amino acids in soil (32), making it a meaningful indicator of amino acid utilization in trees and microbes (5). The glycine cleavage system, one of the main degradation pathways of glycine, has been hypothesized to favor ^15^N and cause the ^15^N enrichment of NH_4_^+^ products (20). Thus, δ^15^N-NH_4_^+^ may be higher than δ^15^N-biomass when the glycine cleavage system is rate-limiting and NH_4_^+^ is excreted from the microbial biomass. Since δ^15^N-NH_4_^+^ was significantly lower than δ^15^N-biomass in the present study (Table S3), we obtained little or no evidence for glycine cleavage.

Collins *et al*. (10) incubated *Escherichia coli* with glycine as the sole N source. The findings obtained demonstrated that δ^15^N-biomass was high in the early stage of the experiments (before 50 h), while δ^15^N-biomass and δ^15^N-NH_4_^+^ approached the δ^15^N of substrates in the later stages (after 50 h) in CN5 media. These findings are in contrast to the present results showing that δ^15^N-biomass increased over time (Fig. 3A), with δ^15^N-NH_4_^+^ being significantly lower than δ^15^N-biomass (Table S3) in CN5 media. These differences were attributed to the presence of NH_4_^+^ re-assimilation or differences in glycine metabolic pathways. In the study by Collins *et al*. (10), NH_4_^+^ re-assimilation, with large isotope fractionation (25), occurred in the later stages (the stationary phase of *E. coli*), such that δ^15^N-biomass decreased and δ^15^N-NH_4_^+^ approached the δ^15^N of substrates. On the other hand, in our experiment, NH_4_^+^ re-assimilation did not occur in the later stages when *A. oryzae* actively mineralized organic N. Furthermore, the glycine cleavage system may have caused the enrichment of δ^15^N-NH_4_^+^ and the depletion of δ^15^N-biomass in the study by Collins *et al*. (10), but not the present study.

### NUE controls ^15^N enrichment

The present results on *A. oryzae* revealed a negative linear correlation between NUE and Δ^15^N (Fig. 4B), indicating the control of N limitations on δ^15^N in the microbial biomass and mineralized NH_4_^+^ under laboratory conditions. Soil microbes may widely change their NUE, while NUE by animals remains low (less than 0.5 [7, 8]), with a constant and large difference (3–4.8‰) between the δ^15^N of animals and substrates (6, 44, 52, 57). Therefore, the present results imply that NUE influences ^15^N enrichment; relatively constant ^15^N enrichment and NUE for animals and variable ^15^N enrichment and NUE for microbes.

These results imply similar control over δ^15^N-SOM patterns in the field; however, further research is needed to test this hypothesis under natural ecosystem settings. δ^15^N-SOM has been shown to markedly change with soil depth, with more substantial ^15^N enrichment being observed from shallow to deep soil layers (2, 24, 43, 48, 50). Since microbially-derived organic N may contribute to SOM (40, 49), the δ^15^N of SOM is also affected by δ^15^N in the microbial biomass, which is controlled by microbial NUE. Further research is warranted to understand the control of soil microbial stoichiometry on δ^15^N-SOM, and the present results suggest a hypothesis that has the potential to link microbial N demands to δ^15^N, thereby offering a non-intrusive and integrative tool through which to understand nutrient limitations in soil decomposer communities.

## Supplementary Information



## Figures and Tables

**Table 1:** Previous findings on differences between δ^15^N-SMB and δ^15^N-SOM (Δ^15^N).

Δ^15^N (‰)(=δ^15^N_SMB_–δ^15^N_SOM_)	*n*=	Depth (cm)	Period	Location	Information	Reference
+0.1 to +0.6	3 for each treatment	0–5	October, 1996	Kansas, USA	8-year treated soils including no chamber, an ambient CO_2_ chamber, and an elevated CO_2_ chamber	Williams *et al*., 2006 (62)
+3.1±0.2	136	A0, 0–10, 0–15	1997 to 2004	Arizona and Florida, USA	six experiments covering a broad range of soil types, vegetation cover, climates, land-use practices, and analytical procedures	Dijkstra *et al*., 2006a (14)
+0 to +4	19	0–10	October 2002 September 2003	Hawaii and Arizona, USA	soils at different elevations	Dijkstra *et al*., 2008 (16)
−1 to +7 (+5.6±0.5)	22	0–10	March, 2003	Arizona, USA	soils in a cattle manure gradient	Dijkstra *et al*., 2006b (15)
+2 to +10 (+7.2±0.7)	31	0–10	March, 2005	northern Arizona, USA	four study sites with different substrate ages of 930 y, 55,000 y, 750,000 y, and 3,000,000 y	Coyle *et al*., 2009 (12)
+0.3±0.3 to +2.2±0.3 (from day 0 to day 180)	3	0–30		Varsailles, France	soil incubation for 6 months	Lerch *et al*., 2011 (41)
−2 to +1	8 for each plot	0–5	April to September, 2007	Kansas, USA	4 plots including fertilized, unfertilized, hayed, and non-hayed	Tiemann and Billings, 2011 (58)
+0 to +8	15	0–10	November, 2014	Danjiangkou Reservoir area, China	soils in woodland plantation, shrubland plantation, cropland, and open areas	Wu *et al*., 2016 (63)
+17	30	thin humus layer	July, 2011	nothern Norway	soils in tundra sites	Barthelemy *et al*., 2017 (1)
