# Supplementary material for: Control of the Nitrogen Isotope Composition of the Fungal Biomass: Evidence of Microbial Nitrogen Use Efficiency
Source: Microbes Environ. 2018 Dec 15;34(1):5–12. doi: 10.1264/jsme2.ME18082 (PMC6440729; doi:10.1264/jsme2.ME18082)
Supplement: Supplementary file 1 [file 34_5_s1.pdf]

Supplemental Material

Controls on the nitrogen isotope composition of fungal biomass: Evidence of microbial nitrogen use efficiency

Kazuki Shinoda[1], Midori Yano[2,3], Muneoki Yoh[2], Makoto Yoshida[2], Akiko Makabe[2,4], Yohei Yamagata[2], Benjamin Z. Houlton[5], Keisuke Koba[2,3*]

[1]United Graduate School of Agricultural Science, Tokyo University of Agriculture and Technology, Tokyo, 183-8509, JAPAN.

[2]Institute of Agriculture, Tokyo University of Agriculture and Technology, Tokyo, 183-8509, JAPAN.

[3]Center for Ecological Research, Kyoto University, Shiga, 520-2113, JAPAN.

[4]Project Team for Development of New-generation Research Protocol for Submarine Resources, Japan Agency for Marine-Earth Science and Technology, Kanagawa, 237-0061, JAPAN.

[5]Department of Land Air and Water Resources, University of California, Davis, California 95616, USA

Corresponding author:
Keisuke Koba
Center for Ecological Research, Kyoto University
keikoba@ecology.kyoto-u.ac.jp

Content:

Table S1. Statistical result of differences between DOC/TDN and biomass C/N.

Table S2. Statistical result of differences between δ^{15}N-biomass at 24 and 96 h.

Table S3. Statistical result of differences between δ^{15}N-NH_4^+ and δ^{15}N-biomass at treatment CN5 and CN10

Table S4. Statistical result of differences betweenδ^{15}N-Gly_{used} and δ^{15}N-glycine (0.1±0.3‰) at treatment CN5 and CN10

Table S5. All data of our study includes changes in weight, pH, DOC, TDN, NH_4^+, DON, NUE, DOC/TDN, biomass C, biomass N, biomass C/N, δ^{15}N-biomass, δ^{15}N-NH_4^+, δ^{15}N-TDN, δ^{15}N-glycine, Δ^{15}N at CN5-100.

Fig. S1. Changes in recovery of N at different C:N treatments.

Fig. S2. A) Relation between DOC/TDN and NUE at treatment CN5 and CN10 and B) Relation between DOC/TDN and NUE at all treatments.

Table S1. Statistical result of differences between DOC/TDN and biomass C/N at each treatment based on Student's *t* test. Bold indicates significant difference (P<0.05).

C/N	Student's *t* test	incubation time			
		24 hr	48 hr	72 hr	96 hr
5	*T*	15.10	13.02	36.63	36.63
	P	**<0.001**	**<0.001**	**<0.001**	**<0.001**
10	*T*	-1.80	0.45	30.25	46.81
	P	0.169	0.673	**<0.001**	**<0.001**
30	*T*	-22.11	-46.60	-14.96	-22.98
	P	**<0.001**	**<0.001**	**<0.001**	**<0.001**
50	*T*	-4.72	-6.98	-11.33	-8.89
	P	**0.009**	**0.002**	**<0.001**	**0.001**
100	*T*	-8.89	-17.88	-4.18	-4.23
	P	**0.001**	**<0.001**	**0.014**	**0.013**

Table S2. Statistical result of differences between δ^{15}N-biomass at 24 and 96 h at each treatment based on paired Student's *t* test, and δ^{15}N-biomass at 96 h and δ^{15}N-Glycine based on Student's *t* test. Bold indicates significant difference (P<0.05).

C/N	paired Student's *t* test		Student's *t* test	
	T	*P*	*T*	*P*
5	-12.90	**0.001**	5.50	**<0.001**
10	-66.30	**<0.001**	5.15	**<0.001**
30	-3.43	0.076	0.95	0.358
50	-5.08	**0.037**	1.47	0.162
100	-1.47	0.279	1.26	0.227

Table S3. Statistical result of differences between $\delta^{15}N\text{-}NH_4^+$ and $\delta^{15}N$-biomass at treatment CN5 and CN10 based on paired Student's *t* test. Bold indicates significant difference ($P<0.05$).

C/N	incubation time (hr)	paired Student's *t* test	
		T	*P*
5	48	13.18	**0.001**
5	72	10.58	**0.002**
5	96	12.38	**0.001**
10	48	180.83	**0.004**
10	72	40.36	**0.001**
10	96	36.23	**0.001**

Table S4. Statistical result of differences between δ^{15}N-Gly$_{used}$ and δ^{15}N-glycine (0.1±0.3‰) at treatment CN5 and CN10 based on paired Student's *t* test. Bold indicates significant difference (*P*<0.05).

C/N	incubation time (hr)	Student's *t* test	
		T	*P*
5	48	-2.37	**0.034**
5	72	-6.77	**<0.001**
5	96	0.24	0.816
10	48	3.51	**0.004**
10	72	1.67	0.119
10	96	-0.13	0.900

Table S5. All data of our study includes changes in weight, pH, DOC, TDN, NH_4^+, DON, NUE, DOC/TDN, biomass C, biomass N, biomass C/N, $\delta^{15}N$-biomass, $\delta^{15}N$-NH_4^+, $\delta^{15}N$-TDN, $\delta^{15}N$-glycine, $\Delta^{15}N$ at CN5-100.

C/N	hr	weight	pH	DOC	TDN	NH4+	DON	NUE	DOC/TDN	biomass C	biomass N	biomass C/N	δ15N-biomass	δ15N-NH4+	δ15N-TDN	δ15N-glycine	Δ15N (= δ15N-biomass – δ15N-glycine)
		g		mgC/vial	mgN/vial	mgN/vial	mgN/vial		mol/mol	mgC/vial	mgN/vial	mol/mol	‰	‰	‰	‰	‰
5	0		5.9	525.81	116.80	0.02	116.78		5.25						0.00	0.00	
5	0		5.7	529.71	111.98	0.02	111.95		5.52						-0.17	-0.17	
5	0		5.9	529.71	112.83	0.02	112.80		5.48						-0.12	-0.12	
5	0		5.9	517.76	111.03	0.02	111.01		5.44						-0.06	-0.06	
5	24	0.12	5.7	378.75	103.88	0.08	103.80	1.00	4.25	34.53	6.55	6.15	-0.18		-0.08		-0.10
5	24	0.13	5.7	354.74	97.58	0.09	97.49	1.00	4.24	40.89	6.98	6.84	-0.21		-0.11		-0.12
5	24	0.04	5.6	364.74	99.03	0.09	98.95	1.00	4.30	12.51	2.23	6.55	-0.11		-0.09		-0.02
5	24	0.11	5.8	341.49	95.93	0.07	95.86	1.00	4.15	34.14	6.24	6.38	0.01		-0.10		0.10
5	48	0.21	6.2	199.47	93.77	19.82	66.16	0.61	2.48	64.61	11.29	6.68	1.20	-1.83	-0.42		1.28
5	48	0.28	6.4	177.70	87.29	23.74	56.45	0.57	2.38	77.74	16.94	5.36	1.20	-3.10	-0.62		1.28
5	48	0.25	6.4	187.40	89.27	23.04	58.93	0.57	2.45	87.19	16.69	6.09	1.38	-2.87	-0.40		1.47
5	48	0.23	6.4	174.39	90.16	25.92	55.98	0.53	2.26	67.18	12.29	6.38	1.06	-2.89	0.29		1.15
5	72	0.32	7.1	23.21	81.20	93.31	0.00	0.20	0.33	94.11	15.13	7.26	1.30	-0.68	-0.27		1.39
5	72	0.29	7.1	17.91	81.16	98.01	0.00	0.12	0.26	91.05	13.25	8.02	2.38	-0.80	-0.58		2.47
5	72	0.29	7.1	22.46	83.08	102.18	0.00	0.09	0.32	92.22	13.16	8.17	2.16	-0.59	-0.65		2.25
5	72	0.29	7.1	15.55	82.26	93.37	0.00	0.16	0.22	89.96	13.71	7.66	2.26	-0.60	-0.62		2.35
5	96	0.35	7.2	14.26	98.14	106.29	0.00	0.09	0.17	122.73	23.13	6.19	2.30	-0.44	-1.00		2.39
5	96	0.38	7.1	15.78	87.99	105.02	0.00	0.06	0.21	132.12	25.22	6.11	3.43	-0.57	-0.71		3.52
5	96	0.36	7.1	16.02	88.91	97.78	0.00	0.13	0.21	124.60	24.22	6.00	3.09	-0.38	-0.88		3.17
5	96	0.38	7.3	15.35	97.93	109.39	0.00	0.01	0.18	129.05	24.22	6.22	3.33	-0.55	-0.84		3.42

C/N	hr	weight	pH	DOC	TDN	NH4+	DON	NUE	DOC/TDN	biomass C	biomass N	biomass C/N	δ15N-biomass	δ15N-NH4+	δ15N-TDN	δ15N-glycine	Δ15N (= δ15N-biomass – δ15N-glycine)
		g		mgC/vial	mgN/vial	mgN/vial	mgN/vial		mol/mol	mgC/vial	mgN/vial	mol/mol	‰	‰	‰	‰	‰
10	0		5.7	519.71	55.09	0.03	55.06		11.01								
10	0		5.9	524.10	55.22	0.03	55.19		11.07								
10	0		5.7	521.91	55.65	0.03	55.62		10.94						0.25	0.25	
10	24	0.04	5.7	410.26	45.40	0.00	45.40	1.00	10.54	11.02	1.58	8.16	-0.41		0.28		-0.65
10	24	0.06	5.7	389.75	44.05	0.05	44.00	1.00	10.32	5.52	2.46	2.62	-0.13		0.26		-0.38
10	24	0.08	5.7	393.75		0.00				20.65	3.49	6.89	-0.13		0.25		-0.38
10	48	0.23	5.5	179.71	26.73	1.91	24.82	0.94	7.84	80.20	10.05	9.31	0.32	-20.59	-0.26		0.07
10	48	0.19	5.7	172.72	28.35	4.02	24.33	0.87	7.11	66.43	8.53	9.08	0.41	-20.27	-0.68		0.16
10	48	0.26	5.8	175.19	28.76	4.84	23.92	0.85	7.11	72.73	15.66	5.42		-22.68	-0.60		-0.25
10	72	0.44	6.5	26.05	21.51	30.35	0.00	0.45	1.41	128.05	21.09	7.08	1.92	-2.51	-2.11		1.67
10	72	0.43	6.4	25.97	23.44	31.60	0.00	0.43	1.29	130.41	20.82	7.31	2.06	-2.75	-2.17		1.82
10	72	0.37	6.4	24.28	23.00	32.52	0.00	0.42	1.23	120.27	18.14	7.74	2.13	-2.40	-2.08		1.88
10	96	0.38	6.5	18.16	27.12	36.72	0.00	0.33	0.78	125.48	22.44	6.52	2.47	-2.24	-1.83		2.22
10	96	0.47	6.6	19.54	27.46	37.12	0.00	0.33	0.83	131.32	24.50	6.25	2.84	-2.34	-2.03		2.59
10	96	0.35	6.6	19.33	28.30	20.47	0.00	0.63	0.80	121.51	21.27	6.66	2.69	-2.22	-1.96		2.44

C/N	hr	weight	pH	DOC	TDN	NH4+	DON	NUE	DOC/TDN	biomass C	biomass N	biomass C/N	δ15N-biomass	δ15N-NH4+	δ15N-TDN	δ15N-glycine	Δ15N (= δ15N-biomass – δ15N-glycine)
		g		mgC/vial	mgN/vial	mgN/vial	mgN/vial		mol/mol	mgC/vial	mgN/vial	mol/mol	‰	‰	‰	‰	‰
30	0		5.7	482.90	18.09	0.02	18.07		31.14						-0.06	-0.06	
30	0		5.8	478.99	18.20	0.02	18.18		30.70						-0.06	-0.06	
30	0		5.7	475.34	17.59	0.02	17.57		31.52						-0.12	-0.12	
30	24	0.03	5.7	400.25	11.39	0.10	10.53	0.99	40.99	1.55	1.13	1.61	-0.10		0.13		-0.02
30	24	0.07	5.8	387.75	10.63	0.09	11.52	0.99	42.56	14.11	2.66	6.19	-0.33		0.27		-0.25
30	24	0.03	5.7	400.00	11.60	0.07	1.64	1.00	40.22	12.19	2.34	6.07	-0.23		-2.87		-0.15
30	48	0.23	5.3	212.09	0.00	0.10	0.00	1.00		71.95	9.30	9.03	0.16		-2.14		0.24
30	48	0.22	5.3	179.04	0.80	0.07	0.72	1.00	262.28	57.98	9.76	6.93	0.05		-1.89		0.13
30	48		5.2	203.96	0.94	0.10	0.85	1.00	251.80						-2.05		0.08
30	72	0.34	5.5	79.87	1.18	0.39	0.79	0.98	78.99	120.10	8.88	15.77	0.88		-1.62		0.95
30	72	0.19	5.7	57.89	0.94	0.22	0.72	0.99	71.77	48.35	6.51	8.67	0.86		-1.61		0.94
30	72	0.42	5.5	70.46	1.22	0.19	1.04	0.99	67.31	121.55	15.72	9.02	0.53		-1.52		0.61
30	96	0.36	5.7	25.44	1.21	0.19	1.02	0.99	24.48	125.60	14.70	9.97	0.17	-23.68	-1.72		0.25
30	96	0.38	5.8	20.09	0.97	0.21	0.76	0.99	24.23	140.56	15.55	10.55	0.52	-6.70	-1.89		0.60
30	96	0.35	5.8	24.49	1.26	0.21	1.04	0.99	22.77	133.10	14.43	10.76	0.38	-14.56	-1.62		0.46

Table S5. continued

C/N	hr	weight	pH	DOC	TDN	NH4+	DON	NUE	DOC/TDN	biomass C	biomass N	biomass C/N	δ15N-biomass	δ15N-NH4+	δ15N-TDN	δ15N-glycine	Δ15N (= δ15N-biomass – δ15N-glycine)
		g		mgC/vial	mgN/vial	mgN/vial	mgN/vial		mol/mol	mgC/vial	mgN/vial	mol/mol	‰	‰	‰	‰	‰
50	0		5.7	400.00	8.58	0.03	8.56		54.37						-0.01	-0.01	
50	0		5.7	403.41	8.89	0.02	8.87		52.93						0.15	0.15	
50	0		5.7	400.24	9.40	0.02	9.39		49.65						-0.11	-0.11	
50	24	0.04	5.8	308.23	1.71	0.06	0.93	1.00	210.76	8.39	1.46	6.73	-0.35		-0.89		-0.36
50	24	0.08	5.7	286.23	0.99	0.07	2.03	0.99	335.78	21.09	3.78	6.51	-0.05		-1.85		-0.06
50	24	0.04	5.8	310.48	2.10	0.08	0.22	0.99	172.25	12.61	2.45	6.00	0.22		-0.75		0.21
50	48	0.08	5.7	223.15	0.75	0.00	0.75	1.00	344.85	25.11	2.80	10.46	-0.31		-1.81		-0.32
50	48	0.13	5.5	202.26	0.86	0.07	0.79	0.99	272.85	36.91	3.82	11.29	-0.33		-1.12		-0.34
50	48	0.05	5.7	224.64	0.59	0.07	0.52	0.99	442.09	16.72	1.81	10.75	-0.08		-1.44		-0.08
50	72	0.16	5.5	150.48	1.38	0.13	1.26	0.99	126.85	50.94	4.19	14.18	0.14		-0.71		0.13
50	72	0.21	5.6	128.24	1.11	0.11	0.99	0.99	135.21	62.11	4.60	15.75	0.10		-0.66		0.09
50	72	0.18	5.6	149.93	1.07	0.15	0.92	0.98	163.97	49.73	4.38	13.24	0.83		-0.75		0.82
50	96	0.27	5.3	103.41	1.42	0.17	1.25	0.98	84.81	110.06	6.65	19.31	0.63	-3.35	1.06		0.62
50	96	0.31	5.3	75.91	1.14	0.11	1.02	0.99	77.87	137.97	6.69	24.06	0.51	-6.87	1.31		0.50
50	96	0.28	5.3	96.58	1.10	0.16	0.94	0.98	102.77	116.72	6.64	20.50	0.79	-6.18	1.01		0.79
C/N	hr	weight	pH	DOC	TDN	NH4+	DON	NUE	DOC/TDN	biomass C	biomass N	biomass C/N	δ15N-biomass	δ15N-NH4+	δ15N-TDN	δ15N-glycine	Δ15N (= δ15N-biomass – δ15N-glycine)
		g		mgC/vial	mgN/vial	mgN/vial	mgN/vial		mol/mol	mgC/vial	mgN/vial	mol/mol	‰	‰	‰	‰	‰
100	0		5.7	651.13	7.12	0.03	7.09		106.63						-3.25	-3.25	
100	0		5.7	647.96	7.26	0.02	7.23		104.17						0.72	0.72	
100	0		5.7	649.66	7.18	0.03	7.14		105.60						0.53	0.53	
100	24	0.09	5.6	539.04	0.45	0.05	0.40	1.00	1402.34	25.89	3.99	7.58	-1.34				-0.68
100	24	0.08	5.7	547.29	0.44	0.08	0.36	0.99	1456.88	20.39	3.48	6.84	0.32				0.99
100	24	0.10	5.7	525.79	0.00	0.06	0.00	1.00		32.23	4.53	8.30	0.23				0.90
100	48	0.12	5.6	472.12	0.87	0.00	0.87	1.01	629.68	44.32	3.00	17.25	-0.19				0.48
100	48	0.11	5.6	489.32	0.76	0.07	0.68	0.99	754.37	35.12	3.18	12.90	0.31				0.98
100	48	0.09	5.5	455.96	0.73	0.00	0.73	1.01	733.49	33.07	2.21	17.46	0.04				0.71
100	72	0.16	5.3	419.25	0.93	0.10	0.83	0.99	523.95	44.64	2.65	19.67	0.66		-3.77		1.32
100	72	0.13	5.3	435.33	0.57	0.09	0.49	0.99	885.28	37.54	2.58	16.98	0.29		-5.55		0.96
100	72	0.18	5.3	416.35	1.16	0.08	1.08	0.99	418.35	57.76	3.42	19.71	0.64		-3.29		1.31
100	96	0.29	5	379.16	0.96	0.10	0.86	0.99	461.04	130.64	5.47	27.89	0.52	3.41	-1.01		1.19
100	96	0.27	5.3	381.87	0.59	0.12	0.46	0.98	755.56	122.76	5.55	25.81	0.50	-5.14	-1.63		1.17
100	96	0.29	5	370.49	1.19	0.15	1.04	0.98	362.21	124.99	5.61	26.00	0.56	-2.45	-0.63		1.23

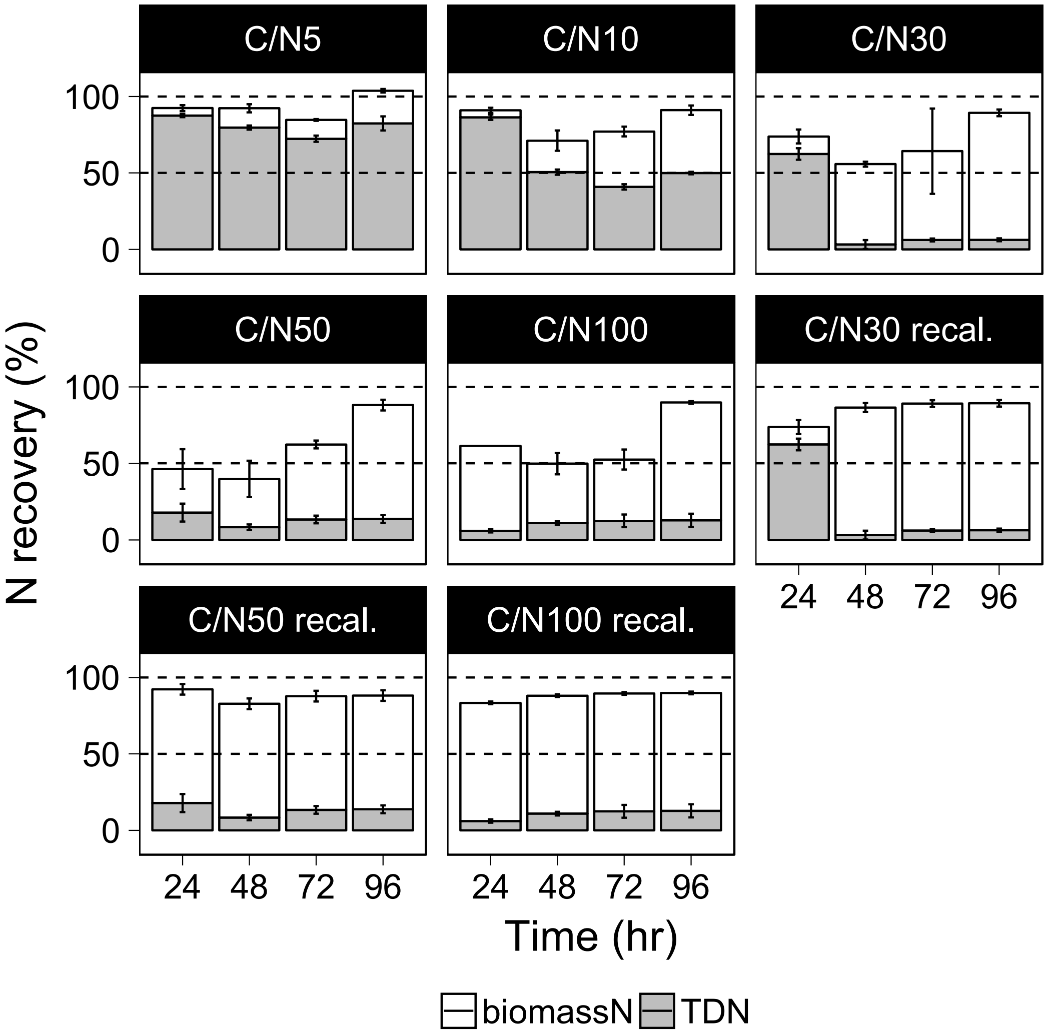

Fig. S1. Changes in recovery of N at different C:N treatments. Gray bars represent recovery of TDN, white bars represent recovery of biomass N and error bars represent SD. C/N 30 recal., 50 recal. and 100 recal. represent results of recalculation for biomass N.

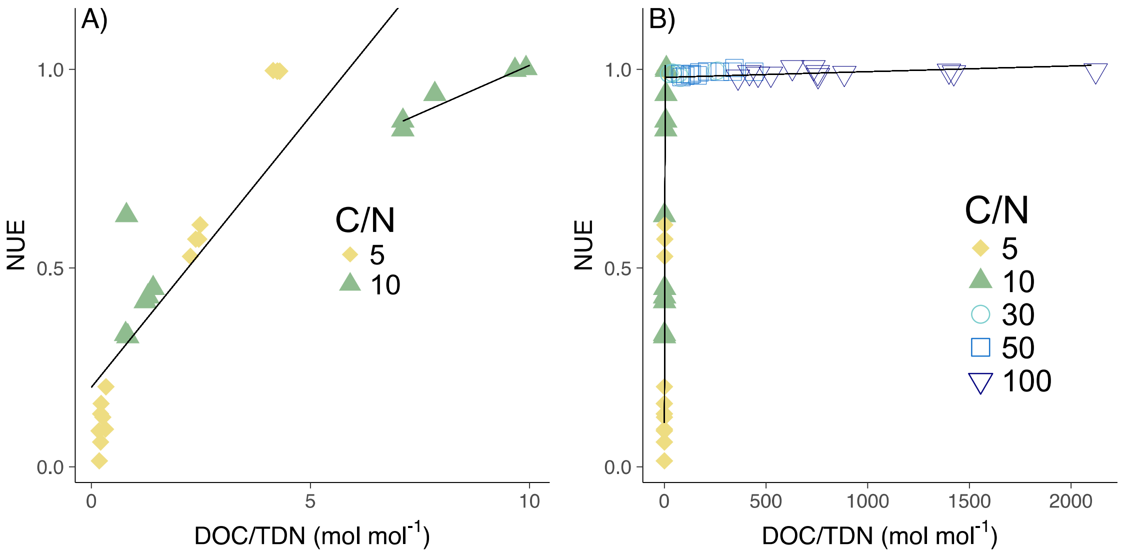

Fig. S2. A) Relation between DOC/TDN and NUE at treatment CN5 and CN10. Black lines represent piece-wise regression line (R^2=0.94, $F_{3,23}$=126.7, P<0.001, n=27). A significant break point was found at a DOC/TDN of 7.11 with a corresponding value of 0.86 for NUE. B) Relation between DOC/TDN and NUE at all treatments. Black lines represent piece-wise regression line (R^2=0.97, $F_{3,58}$=550.8, P<0.001, n=62). A significant break point was found at a DOC/TDN of 4.3 with a corresponding value of 1.00 for NUE.
